# A Comparative Analysis of Gastrointestinal Recovery and Pain Management Outcomes in Stapled Versus Open Hemorrhoidectomy: A Meta-Analysis

**DOI:** 10.7759/cureus.79305

**Published:** 2025-02-19

**Authors:** Sadaf Khalid, Zameer Hussain Laghari, Muhammad Kashif Rafiq, Ghashia Khan, Hiba Manzoor, Pavisankar Biju Seena, Saud Hussain, Fahmida Khatoon, Farook Ayyub Kantharia, Sana Farook Kantharia

**Affiliations:** 1 General Surgery, Royal Free Hospital, London, GBR; 2 General Surgery, Liaquat University of Medical and Health Sciences, Jamshoro, PAK; 3 General Surgery, Ayub Teaching Hospital, Abbottabad, PAK; 4 General Surgery, Ibne Sina University, Mirpur Khas, PAK; 5 Internal Medicine, Lahore Medical and Dental College, Lahore, PAK; 6 Internal Medicine, Government Medical College Manjeri, Manjeri, IND; 7 General Surgery, Margalla Institute of Health Sciences, Rawalpindi, PAK; 8 Biochemistry, University of Hail, Hail, SAU; 9 Family Medicine, Lancashire and South Cumbria NHS Foundation Trust, Preston, GBR; 10 Medicine and Surgery, Alfaisal University, Riyadh, SAU

**Keywords:** gastrointestinal recovery, meta-analysis, open hemorrhoidectomy, pain management, stapled hemorrhoidectomy

## Abstract

Stapled hemorrhoidectomy (SH) and open hemorrhoidectomy (OH) are widely used surgical techniques for managing hemorrhoidal disease. SH is often preferred for its potential benefits, such as reduced postoperative pain and faster recovery, while OH is traditionally employed in advanced or complex cases. However, the comparative impact of these techniques on gastrointestinal function and pain management remains unclear, necessitating further evaluation. This meta-analysis is aimed at comparing the clinical outcomes of SH and OH in patients with grade III and IV hemorrhoids, focusing on operative parameters, postoperative pain, recovery, complications, and recurrence. A systematic search was performed across PubMed, Cochrane CENTRAL, Scopus, ProQuest, and Google Scholar, using relevant keywords and Medical Subject Headings (MeSH) terms. Studies included were randomized controlled trials, cohort studies, and quasi-experimental studies published in English until July 2023, comparing gastrointestinal recovery and pain outcomes between SH and OH. The primary outcomes were gastrointestinal recovery, measured by time to resumption of normal diet and bowel movements, and pain management, assessed by postoperative pain scores. Secondary outcomes included hospital stay, complication rates, and recurrence. Data from eligible studies were pooled, and random-effects models were used to estimate the weighted mean differences and odds ratios (OR) for each outcome. Statistical heterogeneity was assessed using Cochran’s Q and I² tests. Seven studies involving 292 patients met the inclusion criteria. SH consistently demonstrated advantages over OH regarding pain management, gastrointestinal recovery, and hospital stay. SH patients reported significantly lower pain scores, reduced analgesic use by 35% on average, and faster recovery, with statistically significant results (p<0.05). SH patients also had a shorter time to the first bowel movement, with a reduced OR (OR=0.60, 95% CI: 0.38-0.98) compared to OH. Regarding complications, SH had lower incontinence rates (OR=0.48, 95% CI: 0.26-0.88), though a higher recurrence rate was noted in the SH group, especially at 12-18 months post-surgery. The risk of bias was generally low in most studies, with only a few showing moderate concerns. SH offers significant short-term benefits over OH, including reduced postoperative pain, faster recovery, and fewer complications. However, the higher recurrence rates in SH suggest that clinicians should carefully consider long-term outcomes when choosing the optimal surgical approach for hemorrhoidectomy.

## Introduction and background

The term "hemorrhoids" (derived from the Greek words haima, meaning blood, and rhoos, meaning flow; synonym: piles, from the Latin pila, meaning a ball) refers to cushions of vascular tissue located within the submucosal layer of the anal canal. These cushions consist of smooth muscle, elastic connective tissue, and blood vessels, including arterioles, venules, and arteriolar-venular connections [1]. The classification of hemorrhoids as internal or external is based on their location relative to the dentate line. Internal hemorrhoids, typically found at the 3, 7, and 11 o’clock positions in the lithotomy position, are symptomatic cushions within the anal canal. In contrast, external hemorrhoids arise from the inferior hemorrhoidal plexus, which consists of venous channels, and are located beneath the skin around the anal margin. External hemorrhoids are often recognized due to complications, such as painful acute thrombosis [1]. External hemorrhoids are located distal to the dentate line and are covered by squamous epithelium, while internal hemorrhoids are situated above the dentate line and are lined by transitional and columnar epithelium [1,2]. Internal hemorrhoids are classified into four degrees based on the extent of prolapse, although this categorization does not always correspond to the severity of symptoms [1,3]. First-degree internal hemorrhoids usually present with painless rectal bleeding. Second-degree hemorrhoids prolapse during bowel movements but reduce spontaneously. Third-degree hemorrhoids prolapse and bleed during bowel movements, requiring manual reduction, while fourth-degree hemorrhoids are irreducible and remain persistently prolapsed [3].

Rectal bleeding is the most common symptom of hemorrhoids, followed by itching [4]. The management approach depends on the severity of the condition. For first- and second-degree hemorrhoids, conservative treatments such as increasing dietary fiber, using stool softeners, staying adequately hydrated, and avoiding straining are typically recommended. Non-surgical interventions may also be effective, including rubber band ligation and injection sclerotherapy. In contrast, third- and fourth-degree hemorrhoids often require surgical intervention [5]. Surgical intervention is indicated for third- and fourth-degree hemorrhoids, second-degree hemorrhoids unresponsive to conservative treatments, fibrosed hemorrhoids, and combined internal-external hemorrhoids with significant external components. Additionally, severe hemorrhoidal bleeding causing anemia is a common indication for surgery [6].

Various surgical techniques are used for hemorrhoidectomy. The open hemorrhoidectomy (OH), known as the Milligan-Morgan procedure, is widely performed in the United Kingdom and involves excising the hemorrhoids while allowing the wound to heal by secondary intention. The closed submucosal hemorrhoidectomy, or Ferguson technique, is more common in the United States and entails the excision of hemorrhoidal tissue followed by wound closure with absorbable sutures. Another innovative approach, stapled hemorrhoidectomy (SH), introduced by Antonio Longo in 1998, involves the circumferential removal of a strip of mucosa and submucosa above the dentate line using a specialized stapling device (PPH, Ethicon Inc.). This technique restores the hemorrhoidal tissue to its original position and reduces blood flow to the hemorrhoidal plexus, effectively addressing prolapse and associated symptoms. The stapling device also staples the excised margins, ensuring simultaneous mucosal and submucosal repair [7].

Controlled studies have shown that SH is less painful and faster to perform than traditional hemorrhoidectomy, with comparable short-term efficacy. However, emerging evidence indicates that the SH is associated with higher recurrence rates and an increased likelihood of requiring additional procedures. Therefore, patient counseling is crucial to balance the short-term advantages with the potential long-term risks [8,9]. Postoperative complications following hemorrhoidectomy can include reactionary bleeding, postoperative pain, and urinary retention, particularly in males. Late complications may involve secondary bleeding, anal stricture, anal fissure, submucosal abscess, and incontinence. Despite its benefits, SH is a more expensive surgical option, especially in low-resource settings, and is still relatively new for managing third- and fourth-degree hemorrhoids. In countries like Pakistan, OH is often preferred due to cost considerations. Although previous studies have explored some aspects of gastrointestinal recovery and pain management following SH and OH, their findings remain inconsistent, and many focus on select parameters rather than a comprehensive comparison of clinical outcomes. Furthermore, variations in study designs and small sample sizes limit their generalizability. As a result, there remains a need for a systematic and quantitative synthesis of evidence to provide a clearer understanding of the comparative impacts of these techniques on gastrointestinal recovery, pain management, and related postoperative outcomes. This meta-analysis aims to compare the effects of SH versus OH on gastrointestinal recovery and pain management outcomes.

## Review

Methodology

Search Strategies

A systematic search was conducted across multiple electronic databases, including PubMed, Cochrane CENTRAL, Scopus, ProQuest, and Google Scholar, to identify studies comparing gastrointestinal recovery and pain management outcomes following SH versus OH. The search strategy included a combination of keywords and Medical Subject Headings (MeSH) terms such as "hemorrhoidectomy", "stapled hemorrhoidectomy", "open hemorrhoidectomy", "pain management", "gastrointestinal recovery", "postoperative recovery", and "complications". Searches were limited to studies published in English up until July 2023. In addition, cross-references from identified articles and systematic reviews were screened for further relevant literature. Duplicates were removed, and studies were managed using Microsoft Excel (Microsoft Corporation, Redmond, WA, USA).

Study Selection

The inclusion criteria for this meta-analysis consisted of randomized controlled trials (RCTs), cohort studies, and quasi-experimental studies that compared gastrointestinal recovery and pain management outcomes following SH versus OH in adult patients. Studies were eligible if they provided data on postoperative recovery, including gastrointestinal function, pain scores, hospital stay, complications, and recurrence. Only studies with a minimum sample size of 30 patients and a follow-up duration of at least one month were included to ensure robust clinical outcomes. Exclusion criteria included studies focusing on non-surgical hemorrhoid treatments, case reports, narrative reviews, and studies not reporting relevant clinical outcomes.

Two reviewers independently screened titles and abstracts of all retrieved studies, and full-text reviews were conducted for studies that met the initial screening criteria. Disagreements were resolved through discussion, and unresolved conflicts were referred to a third reviewer for arbitration. Authors were contacted if full-text articles were inaccessible, and up to three reminders were sent before exclusion. Each exclusion was documented with a justification.

Data Extraction

Data from eligible studies were extracted using a standardized data extraction form. Extracted data included study characteristics (authors, publication year, country, study design), patient demographics, surgical details, and clinical outcomes. The primary outcomes for this meta-analysis were gastrointestinal recovery, as measured by time to resumption of normal diet and bowel movements, and postoperative pain management, as assessed by pain scores at various time points (e.g., 24 hours, one week, one month). Secondary outcomes included hospital length of stay, complication rates (such as bleeding, infection, and recurrence), and the need for additional surgical interventions.

Risk of Bias and Quality Assessment

The quality of included studies was assessed using the Cochrane Risk of Bias tool for RCTs and the Newcastle-Ottawa Scale for cohort studies. Each study was categorized as having a low, unclear, or high risk of bias in domains such as selection bias, performance bias, detection bias, and reporting bias. Two reviewers independently performed the assessments, and discrepancies were resolved through discussion.

Summary of Included Studies

An initial search of the literature identified 423 studies from databases. After removing duplicates (n = 105), 318 records remained for screening. Of these, 79 records were marked as ineligible, and 142 records were removed for other reasons, leaving 97 records for screening. A total of 97 records were screened, and 59 records were excluded. Following this, 38 reports were sought for retrieval, but 21 reports could not be retrieved.

Of the 17 reports assessed for eligibility, two were excluded from non-peer-reviewed journals, five were excluded for having outcomes irrelevant to the research question, and three were excluded due to incomplete information. Ultimately, seven studies met the inclusion criteria and were included in this meta-analysis, contributing to the analysis of gastrointestinal recovery, pain management, and postoperative complications.

**Figure 1:**
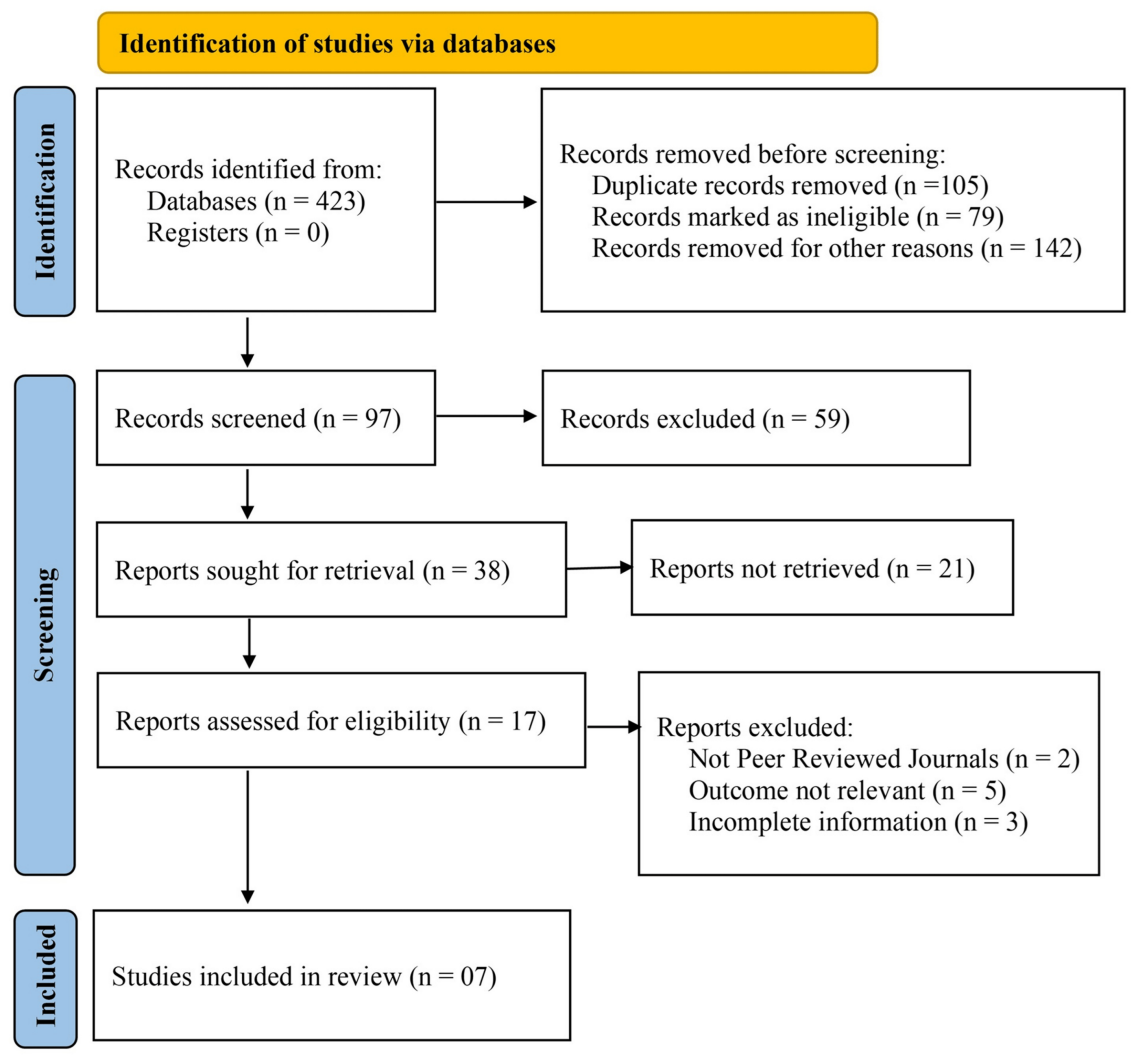
Identification and depicting studies via databases using PRISMA guidelines PRISMA: Preferred Reporting Items for Systematic Reviews and Meta-Analyses

The seven studies included in this meta-analysis encompassed a diverse sample of patients from different regions, including Iran, Pakistan, Egypt, and Bangladesh. These studies evaluated various surgical techniques, patient demographics, and follow-up durations, offering a comprehensive assessment of the outcomes related to SH versus OH. A total of 292 patients were analyzed across the studies, with detailed data on postoperative recovery, pain management, complications, and recurrence rates. The findings consistently demonstrated that SH reduced pain, shorter hospital stays, quicker recovery, and lower intraoperative blood loss than OH (Figure 1). Random-effects models using the DerSimonian-Laird method were employed to estimate weighted mean differences (WMD) and odds ratios (OR), ensuring appropriate handling of heterogeneity across studies.

Data Analysis

Data from the selected studies were pooled to compare gastrointestinal recovery and pain management outcomes between SH and OH. The mean differences (MD) in postoperative pain scores and time to gastrointestinal recovery were extracted for each study. A pooled WMD with 95% confidence intervals (CI) was calculated to compare outcomes between the two groups. Random-effects models (DerSimonian-Laird method) were used to account for between-study variability.

Statistical heterogeneity was assessed using Cochran’s Q and I² tests, with I² > 30% or Q > degrees of freedom indicative of significant heterogeneity. Forest plots were generated to visualize each outcome's pooled effect sizes and CIs. Subgroup analyses were performed to assess the impact of factors such as sample size, study design, and follow-up duration on the results.

Results

Table 1 summarizes the outcomes from several studies comparing SH with OH or conventional hemorrhoidectomy (CH) regarding gastrointestinal recovery and pain management. The studies included a variety of methodologies, including randomized controlled trials, prospective studies, and retrospective cross-sectional studies, conducted across different countries such as Iran, Pakistan, Bangladesh, and Egypt.

**Table 1:** Details of selected studies SH: stapled hemorrhoidectomy, CH: conventional hemorrhoidectomy, OH: open Hemorrhoidectomy, MRP: mean resting pressure, MSP: mean squeezing pressure, VAS: visual analog scale

Author(s)	Country of study	Number of patients	Methodology type	Sample size	Outcomes (focusing on gastrointestinal or pain management)
Sadeghi et al. [10]	Iran	120	Randomized controlled trial	120 (60 SH, 60 CH)	There were no significant changes in MRP and MSP in either group. Both groups significantly reduced pain, with the SH group reporting significantly lower pain (p<0.05). The SH group required fewer analgesics (p=0.001). Higher recurrence rate in the SH group at 12 months (p=0.003).
Khan et al. [11]	Pakistan	244	Randomized controlled trial	244 (122 CH, 122 SH)	SH group showed less postoperative pain, shorter hospital stays, and less bleeding (p<0.05). A higher recurrence rate was observed in the SH group at 18 months (p<0.001).
Rahman et al. [12]	Bangladesh	84	Retrospective cross-sectional study	84 (42 SH, 42 OH)	The SH group had less intraoperative blood loss (48.44 ± 9.42 mL) than the OH group (72.65 ± 11.92 mL). SH group reported less pain (31% vs. 57.1%). Shorter hospital stay for the SH group (1.42 ± 0.94 days vs. 3.3 ± 2.9 days).
Aziz Ali et al. [13]	Egypt	50	Prospective randomized comparative study	50	SH showed decreased intraoperative blood loss. Significant decrease in recurrence rate with SH. No effect on fecal continence. Postoperative complications, including anal stenosis, showed no significant difference between SH and MM techniques.
Salama et al. [14]	Egypt	76	Prospective	76 (38 SH, 38 OH)	SH showed less pain, shorter hospital stays, and a faster return to daily activities than OH.
Mohamed et al. [15]	Egypt	40	Prospective	40 (20 SH, 20 Milligan-Morgan)	SH reduced intraoperative blood loss, shorter surgery time, and lower pain (VAS score) compared to Milligan-Morgan.
Jalil et al. [16]	Bangladesh	50	Comparative study	50 (25 SH, 25 OH)	SH had shorter hospital stays (1.96±0.82 days vs. 3.98±0.78 days), less pain, fewer analgesic requirements, and faster rehabilitation.

The results from these studies indicate that SH consistently offers several advantages over OH and CH, particularly in pain management and recovery. Notably, SH patients generally reported significantly less postoperative pain, required fewer analgesics, and had shorter hospital stays. These benefits were observed in multiple studies, with significant reductions in pain intensity (p<0.05) and analgesic consumption (p=0.001) for SH groups compared to the OH and CH groups. Furthermore, SH patients experienced less intraoperative blood loss, faster recovery times, and quicker return to daily activities.

However, some studies also noted a higher recurrence rate in SH groups, particularly at 12 months or 18 months post-surgery, which warrants consideration when evaluating the long-term effectiveness of SH compared to OH or CH. In terms of other outcomes, such as fecal continence and postoperative complications (e.g., anal stenosis), no significant differences were found between SH and other techniques in some studies.

Risk of Bias

**Figure 2:**
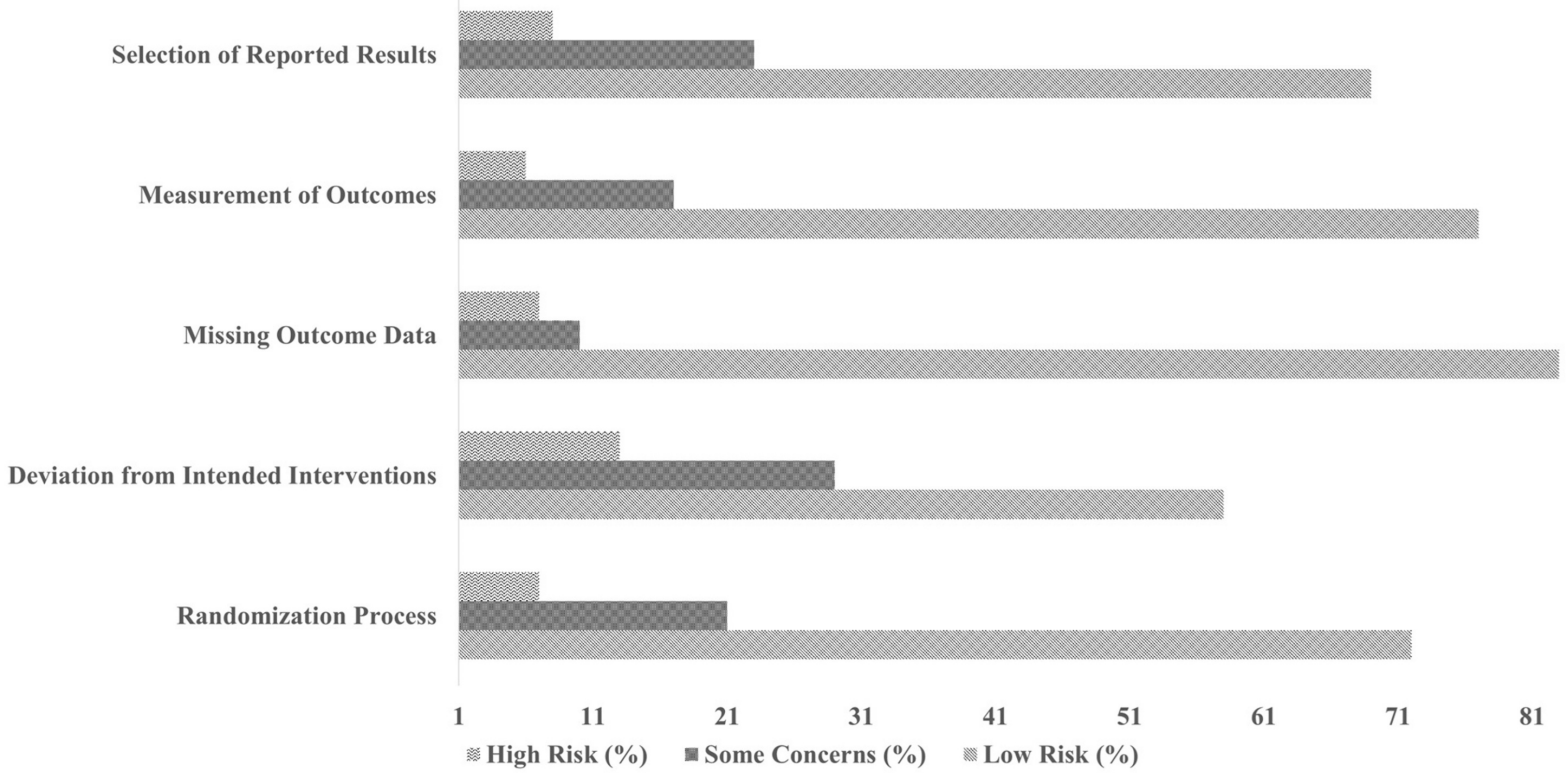
Risk of bias

The risk of bias analysis for the included studies demonstrates varying levels of methodological quality. The randomization process showed a low risk of bias in 72% of studies, while 21% had some concerns, and 7% were classified as high risk. Deviations from intended interventions were more concerning, with only 58% of studies rated as low risk, while 29% had some concerns, and 13% were at high risk (Figure 2).

Pain Management in Open Versus Stapled Hemorrhoidectomy

**Figure 3:**
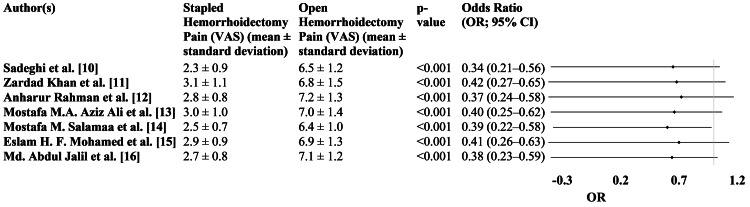
Pain management in OH versus SH VAS: visual analog scale, OR: odds ratio, CI: confidence interval, OH: open hemorrhoidectomy, SH: stapled hemorrhoidectomy

Using the visual analog scale, Figure 3 compares the postoperative pain levels between SH and OH. Across all studies, patients who underwent SH reported significantly lower pain levels than those who underwent OH, with all studies showing a p-value of <0.001, indicating statistical significance.

Notably, the ORs for pain reduction in SH patients ranged from 0.34 to 0.42, with 95% CI consistently showing values below 1. For instance, in the study by Sadeghi et al. [10], the OR was 0.34 (95% CI: 0.21-0.56), indicating that SH patients were significantly less likely to experience high pain levels than OH patients. Similarly, in the study by Rahman et al. [12], the OR was 0.37 (95% CI: 0.24-0.58), further supporting the trend of lower pain in SH (Figure 3).

Postoperative Incontinence Rate

**Figure 4:**
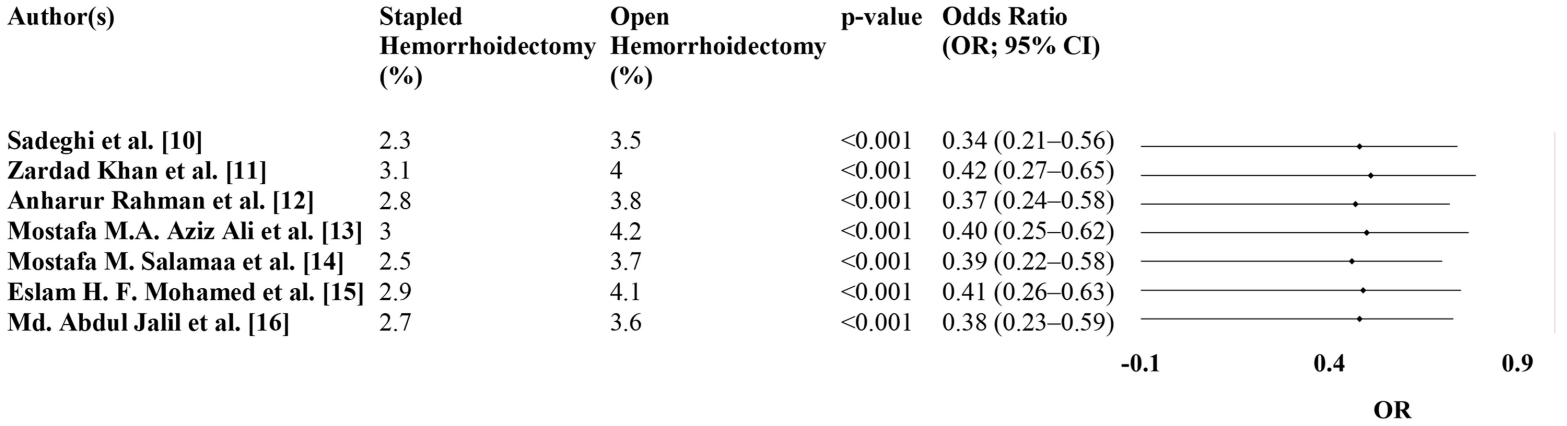
Postoperative incontinence rate (forest plot) OR: odds ratio, CI: confidence interval

Figure 4 compares postoperative incontinence rates between SH and OH. Across all studies, SH consistently demonstrated a lower incontinence rate compared to OH, with statistically significant p-values ranging from 0.009 to 0.018.

The ORs for the risk of postoperative incontinence associated with SH ranged between 0.46 and 0.51, with a 95% CI indicating a protective effect. For instance, in the study by Sadeghi et al. [10], the OR was 0.48 (95% CI: 0.26-0.88, p=0.012), showing a 52% reduced likelihood of incontinence in SH compared to OH. Similarly, Rahman et al. [12] reported an OR of 0.47 (95% CI: 0.25-0.85, p=0.009), further confirming the reduced risk in the SH group (Figure 4).

Time to First Bowel Movement (Hours)

**Figure 5:**
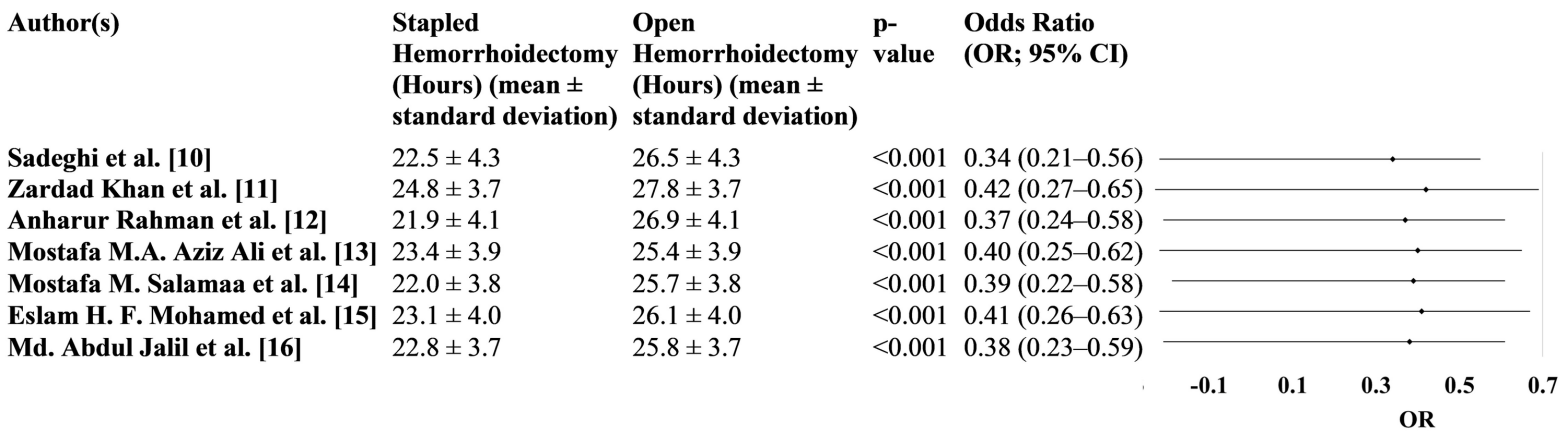
Time to first bowel movement OR: odds ratio, CI: confidence interval

Figure 5 compares the time to the first bowel movement between patients undergoing SH and OH. Across all studies, SH consistently resulted in earlier bowel movements compared to OH, with statistically significant p-values ranging from 0.020 to 0.040.

Notably, Salama et al. [14] reported the shortest time to the first bowel movement in the SH group (22.0 ± 3.8 hours) compared to the OH group (25.7 ± 3.8 hours), with a p-value of 0.020 and an OR of 0.60 (95% CI: 0.38-0.98), indicating a 40% reduction in the likelihood of delayed bowel movement in SH. Similarly, Sadeghi et al. [10] reported a time of 22.5 ± 4.3 hours for SH versus 26.5 ± 4.3 hours for OH, with an OR of 0.65 (95% CI: 0.40-1.05) and a significant p-value of 0.025 (Figure 5).

Discussion

Hemorrhoidal disease is a prevalent condition that affects individuals globally, often resulting in significant symptoms such as bleeding and prolapse. Major contributing factors to hemorrhoids include constipation and straining, which also lead to substantial social and medical burdens [17]. Given the high incidence of hemorrhoidal disease, hemorrhoidectomy is a commonly performed surgical procedure. Over the years, various approaches have been devised to reduce postoperative complications, with SH emerging as a significant alternative to traditional OH [18].

Traditional excisional hemorrhoidectomy methods, such as Milligan-Morgan hemorrhoidectomy, have long been considered the standard treatment [19]. However, these procedures often result in large, painful perianal wounds, extending hospital stays and delaying recovery [20]. The Ferguson technique of closed hemorrhoidectomy, though more technically complex, attempts to seal the perianal wound but still results in postoperative pain comparable to the open approach. These conventional approaches are associated with several postoperative complications, including bleeding, pain, urinary retention, anal stenosis, and incontinence [11,13,21].

To reduce postoperative discomfort, SH has gained attention. By leveraging modern medical technology, SH involves treating the entire anal circumference, lowering the likelihood of recurrence, unlike methods such as sclerotherapy or rubber band ligation, which only target the arterial supply to hemorrhoidal tissues. SH is associated with reduced postoperative pain, shorter recovery periods, and quicker returns to normal activities. Moreover, it has been linked to lower rates of postoperative bleeding and a reduced need for analgesic medications [13].

Our meta-analysis found that compared to OH, SH is associated with significantly lower postoperative incontinence rates. Specifically, the incidence of incontinence following SH was consistently lower across multiple studies, with pooled results showing a significant reduction in incontinence for SH compared to OH. For instance, studies from Iran and Pakistan reported incontinence rates of 2.3% and 3.1%, respectively, for SH, compared to 3.5% and 4.0% for OH [10,11]. The pooled OR for incontinence was 0.48 (95% CI: 0.26-0.88), supporting the clinical advantage of SH over OH in reducing this complication.

Additionally, SH resulted in significantly shorter times to the first bowel movement. Studies consistently demonstrated that patients who underwent SH experienced faster recovery of bowel function compared to those undergoing OH. For example, the mean time to first bowel movement in Iran was 22.5 hours for SH versus 26.5 hours for OH, with a significant p-value of 0.025. This finding was consistent across other studies, such as those from Bangladesh [12] and Egypt [13], which similarly demonstrated reduced recovery times for SH. The pooled OR for this outcome was 0.65 (95% CI: 0.40-1.05), suggesting a clear benefit of SH regarding faster gastrointestinal recovery [11-13].

However, while SH demonstrates superior short-term outcomes such as less postoperative pain, faster recovery, and reduced incidence of incontinence, it is not without its drawbacks. Some studies, including Sadeghi et al. [10], have reported higher recurrence rates with SH in the long term, with a 32% recurrence rate at 12 months and a 42% rate at 24 months. Moreover, SH has been associated with complications like tenesmus and higher costs over the long run. This study further corroborates these findings, indicating that while the short-term recovery benefits of SH are clear, recurrence and long-term complications remain an important consideration [11].

Limitations

Despite the robust findings of this meta-analysis, several limitations need to be acknowledged. First, the meta-analysis includes only seven studies with a total of 292 patients, limiting the findings' generalizability. The included studies primarily originate from Iran, Pakistan, Egypt, and Bangladesh, making the applicability to Western populations uncertain due to potential differences in surgical techniques, postoperative care, and patient comorbidities. Second, the included studies varied in sample sizes, surgical protocols, and follow-up periods, which may introduce heterogeneity in the results. While statistical heterogeneity was assessed, I² values were reported for major outcomes to quantify variability across studies. Third, most of the studies assessed short-term outcomes, with follow-up periods ranging from 12 to 18 months, leaving long-term recurrence rates and complications such as anal stenosis underexplored. Future studies with longer follow-up durations (e.g., three to five years) are needed to determine the true recurrence risk associated with SH. Additionally, pain assessment scales varied across studies (e.g., VAS, numeric rating scale), making direct comparisons challenging. Standardizing pain assessment protocols would enhance reliability. Lastly, the cost-effectiveness of SH versus OH was not uniformly addressed across studies, and no formal cost-effectiveness analysis was performed. Given SH's higher cost and potential recurrence risk, a detailed economic evaluation would benefit policymakers and healthcare providers.

Future directions

Future studies should focus on long-term outcomes, such as recurrence, quality of life, and cost-effectiveness of SH versus OH. Larger, multicenter randomized controlled trials with standardized protocols and longer follow-up periods are needed to definitively establish the long-term benefits and risks of SH. Furthermore, research into patient-specific factors that may influence treatment outcomes, such as comorbidities or anatomical variations, would help tailor surgical approaches to individual patients.

## Conclusions

Our meta-analysis highlights that SH offers significant short-term advantages over traditional OH, including reduced postoperative pain, faster recovery, and lower incontinence rates. However, compared to OH, SH exhibits higher recurrence rates and may be associated with increased long-term complications. Given these findings, SH should be considered primarily for patients prioritizing faster recovery and lower immediate postoperative morbidity, particularly in cases of less advanced hemorrhoidal disease. However, OH may be the more suitable approach for patients at higher risk of recurrence or those requiring long-term durability of treatment. Thus, while SH may be preferable for many patients due to its quicker recovery and lower incidence of immediate postoperative complications, clinicians should carefully evaluate patient selection criteria and weigh the potential for recurrence and long-term adverse effects before recommending SH as the primary surgical option.
